# High Precision Zinc Stable Isotope Measurement of Certified Biological Reference Materials Using the Double Spike Technique and Multiple Collector-ICP-MS

**DOI:** 10.1007/s00216-017-0240-y

**Published:** 2017-02-16

**Authors:** Rebekah E. T. Moore, Fiona Larner, Barry J. Coles, Mark Rehkämper

**Affiliations:** 1 Department of Earth Science and Engineering, Imperial College London, Royal School of Mines, Prince Consort Rd, Kensington London, SW7 2AZ UK; 2 Department of Earth Sciences, University of Oxford, South Parks Road, Oxford, OX1 3AN UK

**Keywords:** Stable isotopes, Zinc, Biological reference materials, Double spike, Multiple collector inductively coupled plasma mass spectrometry, Ion exchange chromatography

## Abstract

Biological reference materials with well-characterised stable isotope compositions are lacking in the field of ‘isotope biochemistry’, which seeks to understand bodily processes that rely on essential metals by determining metal stable isotope ratios. Here, we present Zn stable isotope data for six biological reference materials with certified trace metal concentrations: fish muscle, bovine muscle, pig kidney, human hair, human blood serum and human urine. Replicate analyses of multiple aliquots of each material achieved reproducibilities (2sd) of 0.04–0.13‰ for δ^66/64^Zn (which denotes the deviation of the ^66^Zn/^64^Zn ratio of a sample from a pure Zn reference material in parts per 1000). This implies only very minor isotopic heterogeneities within the samples, rendering them suitable as quality control materials for Zn isotope analyses. This endorsement is reinforced by (i) the close agreement of our Zn isotope data for two of the samples (bovine muscle and human blood serum) to previously published results for different batches of the same material and (ii) the similarity of the isotopic data for the samples (δ^66/64^Zn ≈ –0.8 to 0.0‰) to previously published Zn isotope results for similar biological materials. Further tests revealed that the applied Zn separation procedure is sufficiently effective to enable accurate data acquisition even at low mass resolving power (M/ΔM ≈ 400), as measurements and analyses conducted at much higher mass resolution (M/ΔM ≈ 8500) delivered essentially identical results.

**Electronic supplementary material:**

The online version of this article (doi:10.1007/s00216-017-0240-y) contains supplementary material, which is available to authorized users.

## Introduction

Zinc is an essential biological trace metal as a constituent of hundreds of enzymes with important functions in neurotransmission and for maintaining healthy immune and reproductive systems [1–4]. Disturbances in Zn balance have been linked directly to a number of biological disorders. Tissue Zn mass fractions, hereafter simply referred to as Zn ‘concentrations’, are commonly measured to investigate where and how Zn metabolism varies and whether new early diagnostic tools may be developed to combat diseases associated with such variations [2, 5–15].

A more sensitive way to investigate the metabolism of essential metals is to measure both the metal concentration and stable isotope composition. Such analyses on animal [16–20] and human [21–44] tissue are best performed using multiple collector inductively coupled plasma mass spectrometry (MC-ICP-MS) [45]. With such instruments, it is possible to determine isotope ratios with a precision that is at least an order of magnitude better compared to quadrupole or single collector ICP-MS. This enables the identification of smaller differences in isotope compositions, which in turn can help to identify even very small anomalies in metal metabolism. Despite this, MC-ICP-MS is currently not routinely used in bio-medical research. This may primarily reflect the unfamiliarity of medical researchers with the instrumentation and methods required for high precision isotope analyses and/or the comparatively high cost of the required instruments. In addition, the throughput of methods that are presently in use is severely limited by the time-consuming manual sample preparation, which is required for separation of the analyte element at high yield *and* purity prior to isotopic analysis [46, 47]. The emergence of new techniques to automate and accelerate sample processing [37], however, will help to further establish the value of precise stable isotope measurements for bio-medical investigations.

Laboratories that conduct such research would benefit from the availability of biological materials with well-characterised stable isotope compositions for quality control of analyses, documentation of data quality, and to aid method development and validation. Here, we present high precision Zn isotope data for six commercially available biological reference material (RM) which are certified for concentrations of Zn and other elements [48–53]. The isotopic data were obtained in multiple analyses over a 9-month period. For comparison, an overview of stable Zn isotope compositions for biological materials is also provided. As well as assessing the suitability of these RMs as quality control samples for isotopic research, this study also scrutinised various analytical aspects of current techniques, including material digestion, metal isolation, choice of double spike to sample ratio [54] and MC-ICP-MS mass resolution.

## Materials and methods

### Samples and reagents

Sample preparation was performed in ISO Class 6 metal-free environments with Class 4 laminar flow hoods in the Imperial College London MAGIC Laboratories. AnalaR grade 6 M HCl and 15.4 M HNO_3_ purified in a Teflon still, >18 MΩ cm H_2_O and 30–32% Romil H_2_O_2_ were used throughout. Pre-cleaned Savillex Teflon vials were employed for sample storage throughout all preparation and measurement stages, in order to minimise Zn blanks.

The materials chosen for Zn isotope characterisation were concentration certified, powdered and freeze-dried materials from the Institute of Reference Materials and Measurement (IRMM): ERM-DB001 (human hair), ERM-BB422 (fish (pollock) muscle), ERM-BB184 (bovine muscle) and ERM-BB186 (pig kidney). Also analysed were two batches of Sigma Aldrich BCR-639 (human blood serum), which were stored at −40 °C, and a single batch of concentration certified, freeze-dried Seronorm Trace Elements Urine L-1. The latter RM is distributed in glass vials that contain the dried urine, which is dissolved in 5 ml H_2_O to give certified element concentrations.

The Zn isotope compositions of the samples were determined and are reported relative to a solution of IRMM-3702 Zn, whilst London Zn and JMC Lyon Zn solutions were analysed as secondary reference materials. All samples and standard solutions were doped with a ^64^Zn–^67^Zn double spike solution. This highly enriched tracer is characterised by ^64^Zn/^67^Zn ≈ 2.5 whilst all other Zn isotopes contribute only about 3% to the total Zn budget [55].

### Sample digestion

The powdered IRMM RMs were sampled either four or five times (for pig kidney ERM-BB186), to obtain individual aliquots of between 40 and 60 mg or ∼200 mg. Four 250 μl samples were taken from each batch of human blood serum BCR-639. For the urine, after transferring two of the powder aliquots into Teflon vials and dissolving in 5 ml H_2_O, five 2 ml urine samples were used: two from each diluted aliquot and one mixture of the remaining 1 ml of both. All digests were weighed using a 0.01-mg sensitivity balance.

The samples were broken down by microwave digestion using an Ethos EZ oven, fitted with SK-10 High Pressure Rotor, in acid-cleaned 100 ml Teflon vessels. A blank was included with each set of digestions. A 3:2 mixture of 15.4 M HNO_3_ and H_2_O_2_ was added to each sample to a total of 7 ml for Urine L-1 and 8 ml for all other samples. After 12 h, allowing for partial digestion, the vessels were placed in the microwave oven, which was ramped up to a temperature of 210 °C and held there for 90 min. Following cooling, the sample digests were transferred into Savillex Teflon vials and evaporated to dryness. The hair samples were not fully digested with this method, and an organic residue remained after drying. Full digestion was subsequently achieved by the addition of 15.4 M HNO_3_ and refluxing at 160 °C for 12 h.

### Double spiking

Following evaporation, each digest was dissolved in 2 M HCl to make stock solutions with ∼1 μg/ml Zn (based on the certified concentrations) and aliquots with either ∼250 or ∼800 ng Zn were taken from each. An amount of double spike solution was then added to each aliquot to yield a molar ratio of tracer-derived to natural Zn (S/N) of 1 ± 0.05. The resulting solutions were dried, redissolved in 6 M HCl, left at 130 °C overnight to allow the sample and double spike to equilibrate, and dried again. In addition to this, various London Zn samples were prepared with S/N ratios ranging from ∼0.75 to ∼1.25, to assess the sensitivity of the double spike data reduction to changes in spike to sample ratios. Double spiked solutions of the pure Zn RMs IRMM-3702 Zn, London Zn and JMC Lyon Zn with S/N ≈ 1 were prepared in a similar manner.

### Zinc separation

The double spiked samples were redissolved in 1 M HCl and processed through an established anion exchange chromatography procedure [55, 56] to isolate Zn from the sample matrix. In brief, in-house shrink-fit Teflon columns with resin bed diameters of 3.5 mm were loaded with 250 μl pre-cleaned BioRad AG MP1 100–200 mesh resin. Before sample loading, the resin was cleaned with 2 ml 0.1 M HNO_3_ and 2 ml H_2_O, conditioned with 3 ml 6 M HCl and equilibrated using four aliquots of 0.5 ml 1 M HCl. The samples were loaded onto the resin in 1 ml 1 M HCl. Matrix elements were first removed using 8 ml 1 M HCl and the Zn was then eluted in 6 ml 0.01 M HCl for collection in Teflon vials. After drying, 50 μl 15.4 M HNO_3_ was twice added to each sample and evaporated to dryness, to remove chloride ions and convert the samples into nitrate form for isotope analysis. A London Zn solution was also processed through the ion exchange procedure with each batch of samples for quality control.

### Mass spectrometry

Zinc stable isotope compositions were determined at the Imperial College London MAGIC Laboratory using a Nu Plasma HR MC-ICP-MS and an Aridus II (CETAC Technologies) sample introduction system fitted with glass nebulisers that had typical solution uptake rates of ∼100 μl/min. Faraday collectors L3, Ax, H2 and H4 were used to measure the ion beams of ^64^Zn, ^66^Zn, ^67^Zn and ^68^Zn, respectively, whilst ^62^Ni and ^135^Ba^2+^ (at mass 67.5) were monitored on collectors L5 and H3 to enable corrections for spectral interferences from isobaric Ni and doubly charged Ba ions on Zn isotopes. All collectors were fitted with 10^11^ Ω resistors. Each sample measurement consisted of three blocks of twenty 5 s integration cycles. Electronic baselines were measured for 15 s prior to each block whilst the ion beam was deflected in the electrostatic analyser.

Both low and medium mass resolution were used to measure the Zn isotope ratios of the RMs. Sample and reference material solutions were prepared with 0.1 M HNO_3_ to Zn concentrations of 100 ng/ml or 400 ng/g for measurements at low and medium mass resolution, respectively. At low mass resolution with M/ΔM ≈ 400 (where ΔM is the peak width between 5 and 95% of full peak height), the sensitivity of the instrument was typically ∼120 V/ppm, for a transmission efficiency of ∼0.05%. A medium mass resolution with M/ΔM ≈ 8500 was achieved by adjusting the source defining slit. At such conditions, the sensitivity was typically ∼17 V/ppm, which translates to a much lower transmission efficiency of ∼0.007%.

The Zn isotope compositions are reported as δ^66/64^Zn, expressed in parts per thousand (‰), which was calculated by finding the relative difference between the ^66^Zn/^64^Zn ratios of the sample and a standard (Eq. 1):

1 $$ {\updelta}^{66/64}\mathrm{Z}\mathrm{n}=\left[\left(\frac{{\left({66}_{\mathrm{Zn}}/{64}_{\mathrm{Zn}}\right)}_{\mathrm{Sample}}}{{\left({66}_{\mathrm{Zn}}/{64}_{\mathrm{Zn}}\right)}_{\mathrm{Standard}}}\right)-1\right]\times 1000 $$

Here, IRMM-3702 Zn was used as the δ = 0 standard for all reference material analyses and runs of IRMM-3702 Zn bracketed all sample measurements to enable close monitoring of any drift in instrumental mass bias. The standards solutions that were used for the determination of δ^66/64^Zn values thereby featured S/N values and Zn concentrations, which matched the respective samples within 5 and 10%, respectively. Following acquisition of the ‘raw’ isotopic data, all further data reduction, including instrumental mass bias correction via the double spike [55, 57], corrections for the spectral interferences from Ni^+^ and Ba^2+^ ions, and calculation of the final δ^66/64^Zn values for samples, were carried out offline using an iterative procedure [56, 57]. For quality control, two samples of London Zn were processed and analysed with each batch of RMs, one with anion exchange chromatography and one without, and their δ^66/64^Zn offsets from IRMM-3702 Zn were monitored. The Zn concentrations were calculated offline based on the isotope dilution technique using the ^66^Zn/^68^Zn ratios determined for the sample-spike mixtures in the isotopic runs, following correction for instrumental mass fractionation.

## Results and discussion

### Evaluation of sample preparation procedures

A number of tests were conducted to ascertain the reliability of the sample preparation techniques used in this study for the precise determination of stable Zn isotope compositions. Total procedural Zn blanks ranged between 1 and 6 ng (*n* = 8). Such amounts are essentially negligible, as even the maximum blank would alter the δ^66/64^Zn value of a 250-ng Zn sample by less than 0.02‰, assuming an isotopic offset of 1‰ between blank and sample.

The yield of the anion exchange separation protocol was determined by comparing the expected and observed Zn ion beam intensities during the isotopic measurements and found to be >90% consistently for both the biological RMs and London Zn solutions. In addition, analyses of London Zn samples that were processed by anion exchange chromatography yielded δ^66/64^Zn values that were identical to unprocessed London Zn solutions (Table 1), thereby demonstrating that accurate results are achieved even if the chemical yields are <100%.

**Table 1:** Zn isotope compositions of pure Zn reference materials, relative to IRMM-3702 Zn

Reference material	Reference	δ^66/64^Zn (‰)	2sd	2se	n	m
London Zn	This study: unprocessed^a^	−0.22	0.08	0.04	4	
	This study: processed	−0.20	0.08	0.03	8	5
	This study: all	*−0.20*	*0.07*	*0.02*	*12*	
	Moeller et al. 2012 [58]	−0.19	0.06	0.02	10	
JMC Lyon	This study	−0.32	0.06	0.02	10	
	Archer et al. 2017 [59]	−0.29	0.09	0.01	110	
	Moeller et al. 2012 [58]	−0.30	0.05	0.02	5	
	Van Heghe et al. 2012 [26]	−0.30	0.04	0.01	24	

The 2sd and 2se values were calculated from repeat analyses of the RMs. The total number of measurements for each RM and the number of individual sample aliquots that were processed through separation chemistry are denoted by ‘n’ and ‘m’, respectively

^a^London Zn that was not ‘processed’ through the anion exchange chromatography procedure

The acquisition of accurate results is further supported by the very low levels of spectral interferences from Ni and Ba isotopes, as monitored during analyses of the biological reference samples. In detail, the measurements revealed a typical contribution of 3 ppm ^64^Ni^+^ to the total ion beam at mass 64 and of <1, 5 and 20 ppm for Ba^2+^ ions at masses 66, 67 and 68, respectively. Given these low levels, the applied interference corrections are negligible for all isotopes except ^136^Ba^2+^, where the small correction provides accurate results that are not subject to a systematic bias.

### Results for pure Zn reference materials—London Zn and JMC Lyon Zn

Individual analyses of the Zn standard solutions consistently produced ‘internal’ within-run precisions (2se) of about ±0.05‰ for δ^66/64^Zn. The external, between-run precision (2sd), as determined from multiple analyses of both RMs, was similar or only slightly worse at about ±0.05 to ±0.08‰ (Table 1). The mean δ^66/64^Zn values of London Zn and JMC Lyon Zn, relative to IRMM-3702 Zn, were −0.20 ± 0.07 and −0.32 ± 0.06‰, respectively. These results are fully consistent with previous measurements of different aliquots of these solutions (Table 1).

**Fig. 1:**
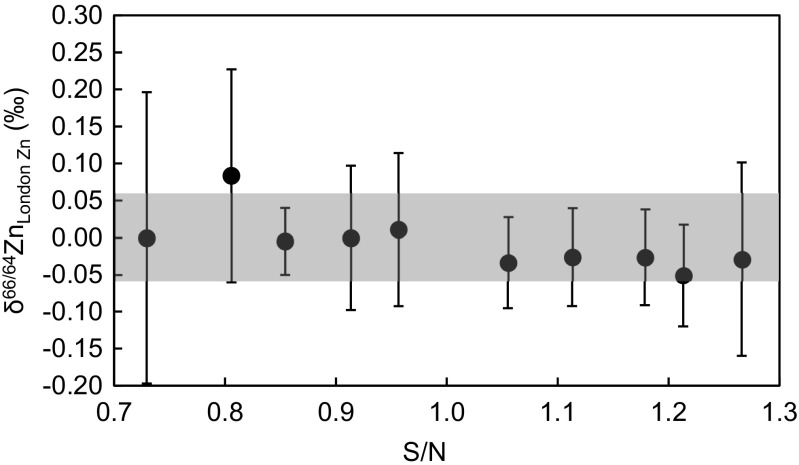
Plot of Zn isotope compositions versus S/N (ratio of spike-derived to natural Zn) for various mixtures of the London Zn reference material with the Zn double spike. All analyses were conducted relative to a London Zn—double spike mixture with S/N = 1 over three measurement sessions and the *shaded field* denotes the mean external precision (2sd) of these analyses. The *error bars* for the individual samples show the 2sd precision determined from multiple sample measurements (*n* = 2–3)

London Zn solutions that were spiked to obtain variable S/N values from about 0.75 to 1.25 and analysed relative to a London Zn solution with S/N ≈ 1 revealed no clearly resolvable deviations from δ^66/64^Zn = 0.00 ± 0.06‰ (Fig. 1). The data, however, suggest a possible trend of decreasing δ^66/64^Zn with increasing S/N. This trend is less apparent if the individual run results for the different S/N ratios are plotted rather than the mean δ^66/64^Zn values obtained from the replicate measurements, particularly because the analytical precision is generally worse for samples with larger deviations from S/N = 1 (see Electronic Supplementary Material, ESM). Given these results, samples are best analysed relative to δ = 0 standard solutions, which feature S/N ratios that match to within about 15% or better (Fig. 1).

### Results for biological reference materials

**Fig. 2:**
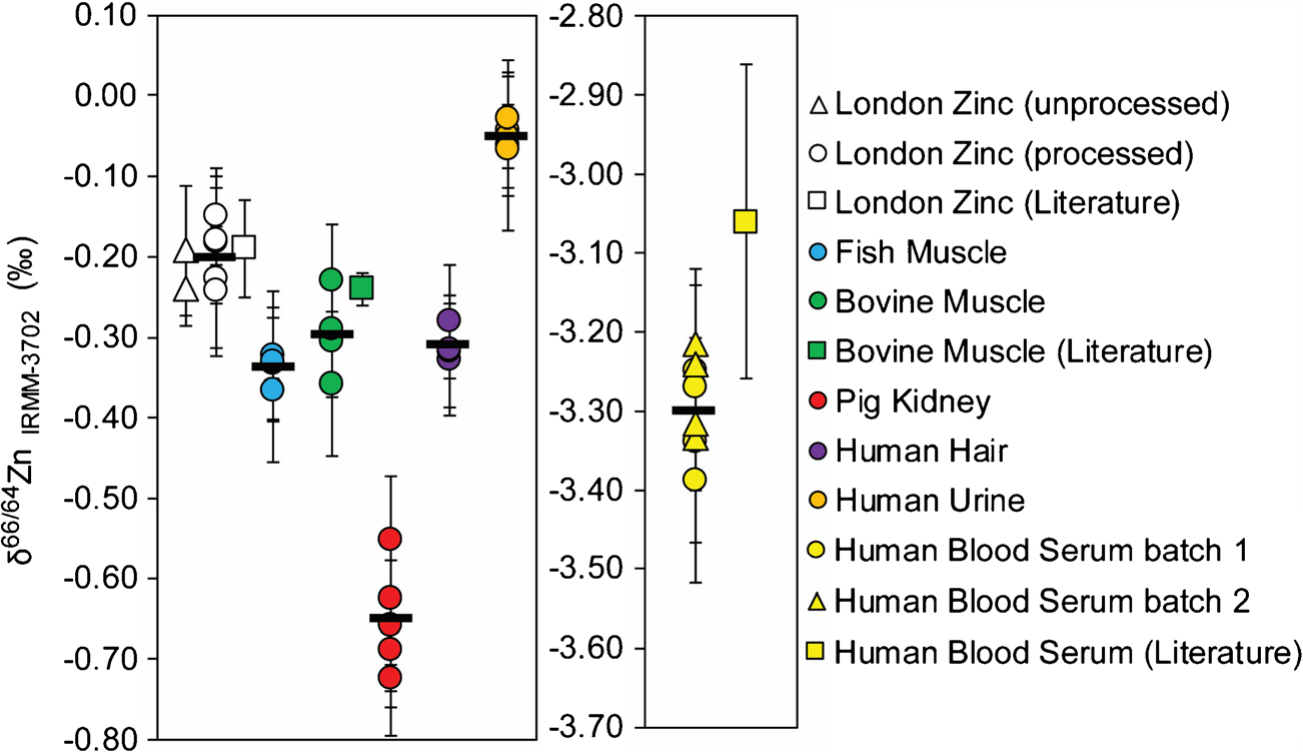
Zinc isotope composition measured for individual digests of the RMs. Each data point denotes the mean of at least two samples from the same digest and *error bars* show the 2sd precision calculated from the individual measurements. The two different London Zn data points from this study represent samples that were ‘processed’ through our anion exchange chromatography procedure and those that were not (unprocessed). For the human blood serum, the results are plotted on a different *y*-axis, due to the large isotopic offset. The δ^66/64^Zn literature data for London Zn, bovine muscle and human blood serum are from Moeller et al. 2012 [58], Costas-Rodríguez et al. 2014 [22] and Larner et al. 2015 [21], respectively

The Zn concentration and stable isotope data that were obtained for the biological RMs are summarised in Table 2 and Fig. 2, together with the certified Zn abundances and any reference values from previous studies.

**Table 2:** Zn concentrations and isotope compositions of six biological RMs

Sample/data source	δ^66/64^Zn_IRMM-3702_ (‰)	±2sd	±2se	n	m	[Zn] ±1sd (μg/g)	Digest weight (mg)
ERM-BB422—fish muscle							
Digest 1	−0.37	0.08		2	1	16.7	62
Digest 2	−0.32	0.08		2	1	15.9	196
Digest 3	−0.33	0.07		2	1	15.4	230
Digest 4	−0.33	0.07		2	2	16.2	212
Mean	−0.34	0.04	0.01	8	5	16.0 ± 0.5	
Certificate [50]						16.0 ± 1.1	
ERM-BB184—bovine muscle							
Digest 1	−0.36	0.08		4	2	152.0	66
Digest 2	−0.31	0.07		2	2	151.0	63
Digest 3	−0.23	0.08		4	2	151.3	213
Digest 4	−0.29	0.06		4	2	152.6	243
Mean	−0.30	0.10	0.03	14	8	151.5 ± 0.4	
Reference [22]	−0.24	0.02					
Certificate [48]						146 ± 7.0	
ERM-BB186—pig kidney							
Digest 1	−0.73	0.07		2	2	144.0	42
Digest 2	−0.69	0.08		4	3	144.0	56
Digest 3	−0.63	0.08		2	1	144.9	195
Digest 4	−0.66	0.08		2	1	145.2	218
Digest 5	−0.55	0.08		2	1	144.6	204
Mean	−0.65	0.13	0.04	12	8	144.5 ± 0.7	
Certificate [49]						134 ± 5	
ERM-DB001—human hair							
Digest 1	−0.33	0.07		3	2	197.4	53
Digest 2	−0.32	0.07		3	2	201.9	43
Digest 3	−0.32	0.07		3	2	203.2	238
Digest 4	−0.28	0.07		3	2	211.8	204
Mean	−0.31	0.04	0.01	12	8	203.6 ± 5.9	
Certificate [52]						209 ± 12.0	
Trace elements urine—L-1							
Digest 1—vial 1	−0.05	0.07		3	2	0.216	2020
Digest 2—vial 1	−0.06	0.11		2	2	0.233	1997
Digest 3—vial 2	−0.05	0.02		2	2	0.214	1870
Digest 4—vial 2	−0.07	0.06		1	2	0.218	2018
Digest 5—vial 1&2	−0.03	0.06		1	1	0.221	1995
Mean	−0.05	0.03	0.01	9	9	0.221 ± 0.008	
Certificate [53]						0.347 ± 0.035	
BCR-639—human blood serum							
Digest 1—batch 1	−3.25	0.08		2	1	2.43	242
Digest 2—batch 1	−3.34	0.08		2	1	2.55	250
Digest 3—batch 1	−3.39	0.08		1	1	2.43	250
Digest 4—batch 1	−3.27	0.08		1	1	2.27	246
Digest 5—batch 2	−3.22	0.08		1	1	2.43	245
Digest 6—batch 2	−3.24	0.08		1	1	2.53	248
Digest 7—batch 2	−3.33	0.10		1	1	2.37	266
Digest 8—batch 2	−3.32	0.10		1	1	2.29	227
Mean	−3.29	0.12	0.04	10	8	2.41 ± 0.10	
Reference [21]	−3.06	0.20					
Certificate [51]						2.36 ± 0.14	

The 2sd data for the δ^66/64^Zn values of individual digest are based on repeat analyses of the matching standard, whereas the 2sd and 2se for the mean δ^66/64^Zn values of each RM (italicised) are calculated from all individual RM measurements. The total number of measurements and the number of individual sample aliquots that went through the separation chemistry for each digest and RM are denoted by ‘n’ and ‘m’, respectively

### Concentration measurements

Overall, the measured Zn concentrations of the biological RMs agree with the official certified values within the combined 1sd uncertainties for all samples except the pig kidney ERM-BB186 and the human urine (Trace Elements Urine L-1) (Table 2). For the pig kidney, our analyses provide a slightly higher Zn content of 144.5 ± 0.7 μg/g compared to the certified concentration of 134 ± 5 μg/g. In the case of the urine, we find a significant deviation from the certified Zn concentration, with a result of 221 ± 8 ng/g versus a reference value of 347 ± 70 ng/g. It is improbable that this difference is primarily due to incomplete transfer of the sample powder from the glass storage vials prior to initial sample dissolution in 5 ml H_2_O (the volume at which the Zn concentration is certified [53]). This is reinforced by the observation that essentially identical Zn concentrations were measured for two different glass vials of the urine RM even though the amount of dry residue recovered from each vial differed slightly (at 136.36 and 126.43 mg). Hence, it is more likely that the low Zn concentration determined here reflects either sample heterogeneity amongst different aliquots or batches of the urine RM or a high Zn blank during the original certification measurements.

### Isotope analysis

For the isotopic analyses of the biological RMs, the internal precision was similar to results obtained for pure Zn solutions at about ±0.05‰ (2se) for δ^66/64^Zn. A similar result emerges for the external reproducibility of data obtained for single digests of the biological RMs that were analysed multiple times and where some digests were split into two or three aliquots for separate processing through the column chemistry (Table 2). In detail, these measurements yielded precisions (2sd) of ±0.04 to ±0.10‰, nearly identical to the reproducibility seen for multiple London Zn and JMC Lyon Zn analyses (Table 1). Together, these results demonstrate that the separation procedure produces clean Zn cuts, which enable precise isotopic analyses that are not affected by spectral or non-spectral interferences.

The accuracy of the Zn stable isotope data can be evaluated, as reference values from previous studies are available for two of the materials, bovine muscle ERM-BB184 and human blood serum BCR-639 (Table 2, Fig. 2). For the bovine muscle, our δ^66/64^Zn result of −0.30 ± 0.10‰ agrees with data from a different laboratory (−0.24 ± 0.02‰ [22]). For BCR-639, the average δ^66/64^Zn value found was −3.29 ± 0.12‰, with no difference between the two batches (−3.31 ± 0.13 and 3.28 ± 0.11‰). The δ^66/64^Zn value of this sample is identical to previous analyses of the RM, which were conducted by a different analyst on a separately purchased batch of the material [21]. Notably, these latter measurements yielded a comparatively large uncertainty of ±0.2‰ (Table 2).

The δ^66/64^Zn data that were obtained for the remaining four reference materials showed only limited variability, with results of –0.34 ± 0.04‰ for fish muscle ERM-BB422, –0.65 ± 0.13‰ for pig kidney ERM-BB186, –0.31 ± 0.04‰ for human hair ERM-DB001 and −0.05 ± 0.04‰ for the Seronorm Trace Elements Urine L-1 (Table 2, Fig. 2). Notably, the dried residue in two glass vials of the urine RM produced solutions with identical Zn isotope compositions. The latter result suggests that the urine RM is likely to have a homogeneous Zn isotope composition despite possible significant differences in Zn concentration.

**Fig. 3:**
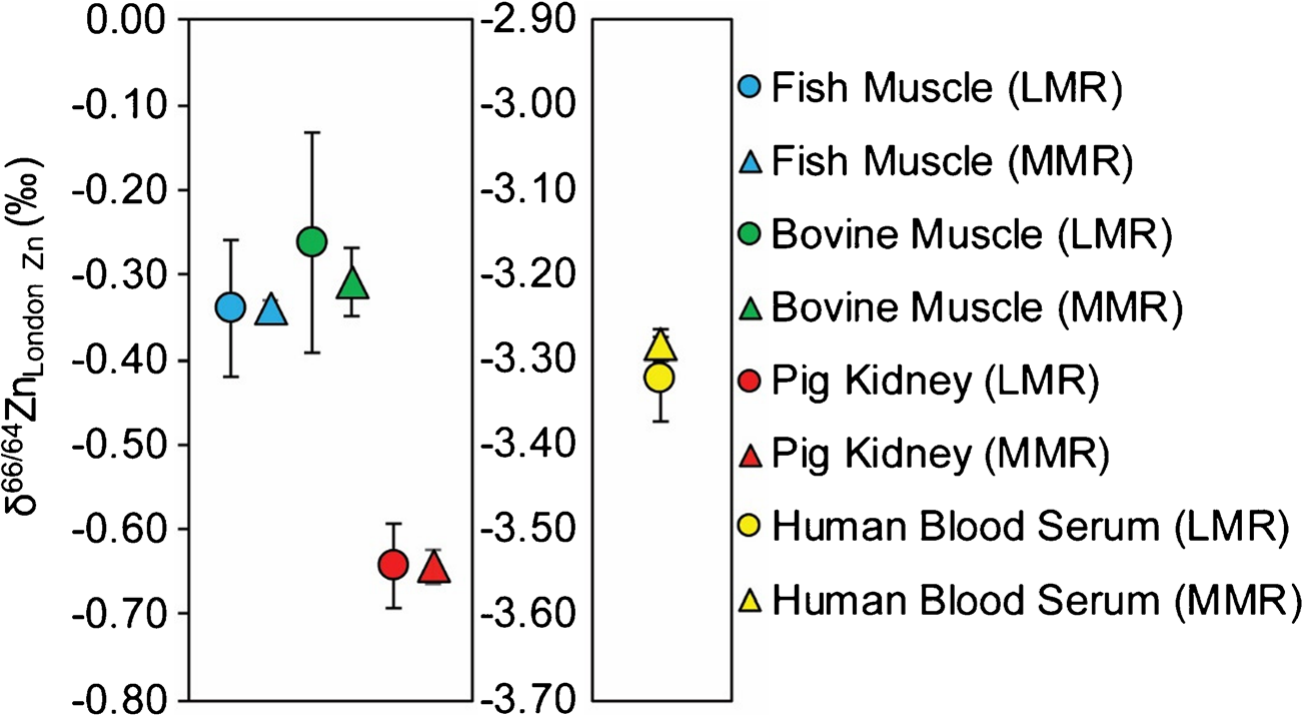
Average δ^66/64^Zn values of RMs obtained in measurements at low and medium mass resolution (*LMR* and *MMR*). Each data point is the mean of six to eight individual measurements and *error bars* represent the 2sd precision

The isotopic analyses for four of the biological RMs were carried out both at low and medium mass resolutions to better ascertain the accuracy of the data. The results for the different analysis modes are compared in Fig. 3. Importantly, this diagram reveals no significant differences between these two sets of data. This indicates that our sample preparation procedure produces Zn solutions, which are sufficiently pure for accurate δ^66/64^Zn measurements even at low mass resolution. Importantly, this allows for more repeat analyses of individual samples or smaller sample size, as much less Zn is needed for a single measurement at low mass resolution than at medium mass resolution, where ion beam transmission is much lower. This may prove important in bio-medical studies because tissue sample size is often limited and sampling is usually only possible on a single occasion.

The overall mean values that were calculated for the six biological RMs are based on at least eight individual analyses, which were obtained on four to eight separate digests. Notably, the 2sd uncertainties of the mean results vary between ±0.04 and ±0.13‰ (Table 2). In detail, three of the biological RMs reveal overall mean results with uncertainties of ≥0.10‰ and which are hence slightly but noticeably larger than the typical 2sd reproducibilities for multiple measurements of a single digest (generally ≤0.08‰). It is possible that this small discrepancy reflects minor sample heterogeneities, which are just noticeable when sampling occurs at the 50 to 200 mg scale. However, given the current uncertainty of stable Zn isotope analyses of about ±0.05 to ±0.15‰ (2sd), this slight heterogeneity will have only a minor impact on any results.

**Fig. 4:**
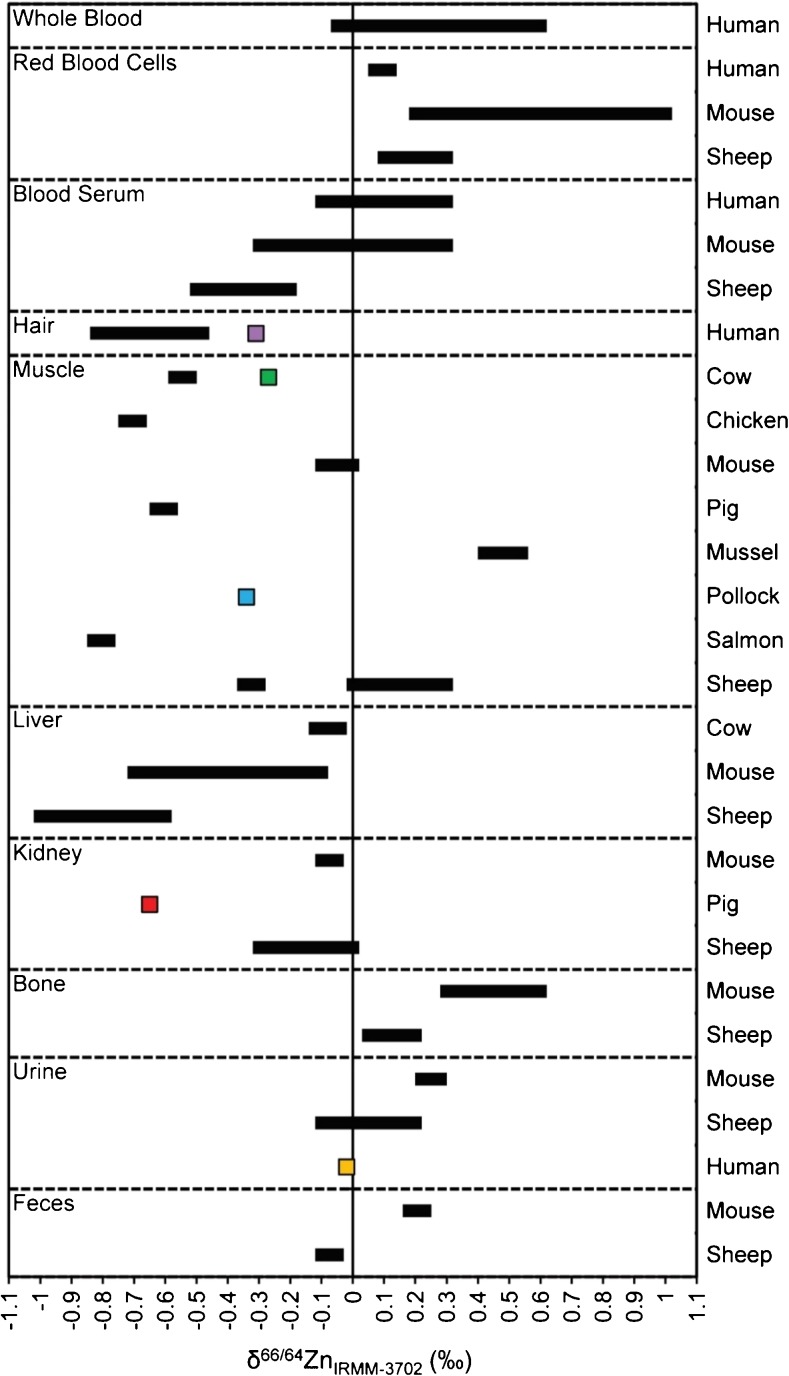
Compilation of published δ^66/64^Zn values for animal tissue and the reference materials analysed in this study (squares, except BCR-639), reported relative to IRMM-3702 Zn [16, 18, 21, 22, 25, 26, 29, 60–62]. Literature data reported relative to JMC Lyon Zn was corrected for an offset of 0.32‰ between IRMM-3702 Zn and JMC Lyon Zn (Table 1)

Also of note is the observation that all but one of the biological RMs have δ^66/64^Zn values of between about ±0.0 and –0.8‰ and which hence fall within the range of stable isotope compositions that were previously measured for such tissues (Fig. 4). As such, they are particularly well-suited as quality control materials for Zn isotope analyses. The exception is BCR-639, which is about 3‰ lower in δ^66/64^Zn compared to previous results obtained for human blood and blood serum (Fig. 4). This large discrepancy is most likely related to the preparation of this RM, which involved the addition of a Zn solution (NIST SRM 3168a) to natural blood serum [63]. It is also conceivable that this method of preparation may be responsible for the possible minor isotopic heterogeneity of this sample, as is indicted by the relatively poor reproducibility of our overall mean result and the comparatively large difference between the data obtained here and previously by Larner et al. [21].

## Conclusions

A double spike technique in conjunction with MC-ICP-MS was used to measure the Zn isotope compositions of six biological reference materials with certified trace metal concentrations. It is shown that these methods are suitable for the routine determination of δ^66/64^Zn with an external precision (2sd) of about ±0.05 to ±0.10‰. Analyses of multiple digests of the biological RMs yielded mean δ^66/64^Zn values with a reproducibility of between ±0.04 and ±0.13‰. This suggests that these RMs feature no or only very minor heterogeneities in Zn isotope composition, when sampling occurs on the scale of 50 to 200 mg. As such, all are in principle ideally suited as quality control materials for Zn isotope measurements in bio-medical research. This conclusion is reinforced by the observations that all samples, except for human blood serum BCR-639, also have Zn isotope compositions that fall within the range of normal natural values. The unusually low δ^66/64^Zn value of BCR-639 reflects that this sample was produced by the addition of a purified Zn solution to natural blood and this non-natural origin may limit the suitability of this material for some applications. Further measurements revealed that isotopic analyses of the samples at low and medium mass resolution produced essentially identical Zn isotope data. This demonstrates that the sample preparation procedure yields sufficiently pure separates of Zn, such that the isotopic measurements do not require medium mass resolution to resolve remaining spectral interferences. This result is advantageous, as low mass resolution analyses require significantly less Zn than measurements at higher mass resolving power.

## Electronic supplementary material

Below is the link to the electronic supplementary material.

ESM 1 (PDF 101 kb)
