# Supplementary material for: High Precision Zinc Stable Isotope Measurement of Certified Biological Reference Materials Using the Double Spike Technique and Multiple Collector-ICP-MS
Source: Anal Bioanal Chem. 2017 Feb 16;409(11):2941–50. doi: 10.1007/s00216-017-0240-y (PMC5366171; doi:10.1007/s00216-017-0240-y)
Supplement: Supplementary file 1 — (PDF 101 kb) [file 216_2017_240_MOESM1_ESM.pdf]

Analytical and Bioanalytical Chemistry

Electronic Supplementary Material

High Precision Zinc Stable Isotope Measurement of Certified Biological Reference Materials Using the Double Spike Technique and Multiple Collector-ICP-MS

Rebekah E. T. Moore, Fiona Larner, Barry J. Coles, Mark Rehkämper

Supplementary figure showing all measurements used to calculate the mean and 2sd values shown in figure 1 (main text)

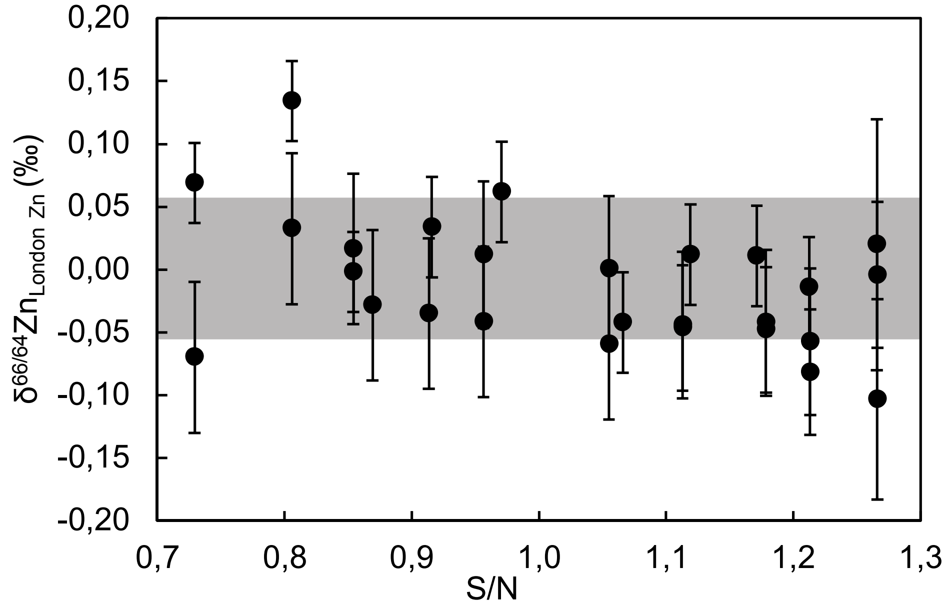

Fig. S1 Plot of Zn isotope compositions versus S/N (ratio of spike-derived to natural Zn) for various mixtures of the London Zn reference material with the Zn double spike. All analyses were conducted relative to a London Zn – double spike mixture with S/N = 1 over three measurement sessions and the shaded field denotes the mean external precision (2sd) of these analyses. The error bars for the individual samples show the 2sd precision determined from repeats of the standard
